# Targeting hypoxic microenvironment of pancreatic xenografts with the hypoxia-activated prodrug TH-302

**DOI:** 10.18632/oncotarget.9654

**Published:** 2016-05-29

**Authors:** Ines Lohse, Joanna Rasowski, Pinjiang Cao, Melania Pintilie, Trevor Do, Ming-Sound Tsao, Richard P. Hill, David W. Hedley

**Affiliations:** ^1^ Ontario Cancer Institute and Campbell Family Cancer Research Institute, Princess Margaret Cancer Center, University Health Network, Toronto, Ontario, Canada; ^2^ Department of Pathology, University Health Network, Toronto, Ontario, Canada; ^3^ Department of Laboratory Medicine and Pathobiology, University of Toronto, Toronto, Ontario, Canada; ^4^ Department of Medical Biophysics, Toronto, Ontario, Canada; ^5^ Department of Radiation Oncology, Toronto, Ontario, Canada; ^6^ Department of Radiation Medicine Program, Toronto, Ontario, Canada; ^7^ Department of STTARR Innovation Center, Toronto, Ontario, Canada; ^8^ Department of Medicine, University of Toronto, Toronto, Ontario, Canada; ^9^ Department of Medical Oncology and Haematology, Princess Margaret Cancer Centre, Toronto, Ontario, Canada

**Keywords:** pancreatic cancer, tumor-initiating cells, hypoxia, TH-302, patient-derived xenograft

## Abstract

Previous reports have suggested that the hypoxic microenvironment provides a niche that supports tumor stem cells, and that this might explain clinical observations linking hypoxia to metastasis. To test this, we examined the effects of a hypoxia-activated prodrug, TH-302, on the tumor-initiating cell (TIC) frequency of patient-derived pancreatic xenografts (PDX).

The frequencies of TIC, measured by limiting dilution assay, varied widely in 11 PDX models, and were correlated with rapid growth but not with the levels of hypoxia. Treatment with either TH-302 or ionizing radiation (IR), to target hypoxic and well-oxygenated regions, respectively, reduced TIC frequency, and the combination of TH-302 and IR was much more effective in all models tested. The combination was also more effective than TH-302 or IR alone controlling tumor growth, particularly treating the more rapidly-growing/hypoxic models. These findings support the clinical utility of hypoxia targeting in combination with radiotherapy to treat pancreatic cancers, but do not provide strong evidence for a hypoxic stem cell niche.

## INTRODUCTION

Hypoxia probably occurs to some extent in all solid tumors when oxygen consumption exceeds supply. It has long been recognized that cells become hypoxic as they are pushed away from blood vessels by the proliferation of oxygenated cells. Although necrosis eventually occurs, chronically hypoxic cells are of clinical importance as they are relatively insensitive to radiotherapy and chemotherapy [1-4] yet capable of regrowth if reoxygenation occurs following the death of the oxic population. It is now recognized that hypoxia has wide-ranging effects, including the increased potential for invasion and metastasis that are not explained by this simple model [5-12]. Consequently there is current interest in the effects of acute (or intermittent) hypoxia, due to temporal fluctuations in blood flow, and in adaptive mechanisms that lead to hypoxia tolerance and thus the accumulation of hypoxic cells. Tumor hypoxia is a dynamic process with major effects on cancer biology, of considerable current interest.

The hypoxic microenvironment of solid tumors comprises a complex interaction between cancer cells and stromal elements, and it has been proposed that this provides a niche supporting the maintenance of cancer cells with stem cell-like features [13-23]. The presence of such cells has been described in pancreatic cancers [14, 16, 17], and they are reported to show distinctive features such as the re-expression of embryonic developmental pathways and a more mesenchymal, invasive phenotype. They do not however show a consistent phenotype that can be used to identify and enumerate them with confidence, and their frequency is functionally defined using limiting dilution assays (LDA). We reasoned that if tumour-initiating cells (TIC) reside in a hypoxic microenvironment, then they would be sensitive to the hypoxia-activated prodrug TH-302 but relatively resistant to ionizing radiation. TH-302 (evofosfamide) is a 2-nitroimidazole [24-27] whose reductive metabolism produces an alkylating species that causes DNA damage in quiescent as well as proliferating cells. It was recently tested in combination with gemcitabine in a large randomized clinical trial, MAESTRO, treating patients with advanced pancreatic cancer. There was a trend towards improved survival in patients treated with the combination, but this did not achieve statistical significance (NCT01746979). However, it should be noted that patients were not stratified based on tumor hypoxia, and the inclusion of patients with low hypoxia tumors might have weakened statistical power. In contrast to other alkylating agents, TH-302 is relatively non-toxic towards well oxygenated normal tissues [24-26]. The effects of TH-302 on tumor growth and TIC frequency were examined as a single agent, and in combination with ionizing radiation which selectively kills oxygenated cells.

## RESULTS

### Characterization of the patient-derived pancreatic xenograft models

**Figure 1:**
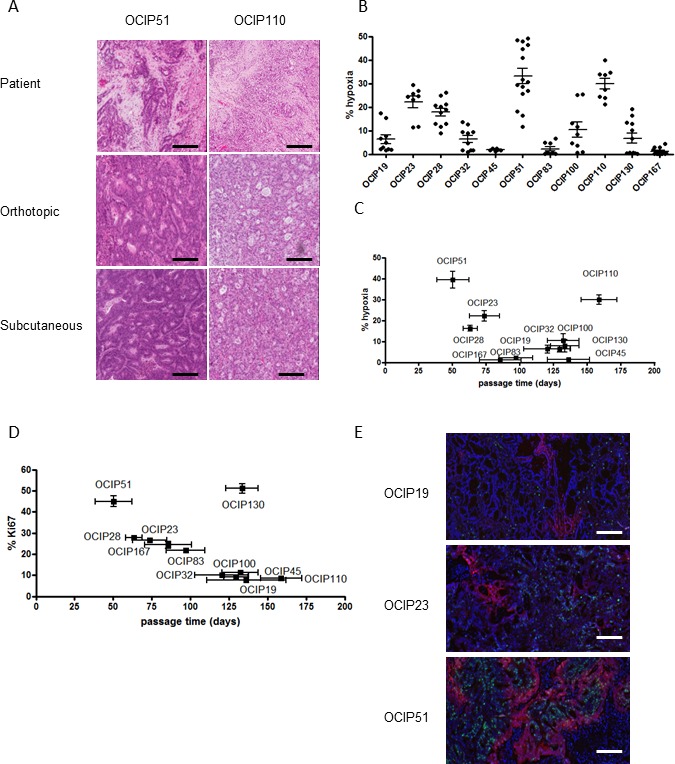
Patient-derived pancreatic xenograft models display different growth rates and hypoxia level and closely resemble the patient tumor **A.** Representative H&E staining of the patient surgical specimen and tumors derived from the orthotopic and subcutaneous site of OCIP51 and OCIP110 show stable tumor morphology after implantation at either site if compared to the patient tumor (scale bars = 100micron). **B.** Percentage of tumor hypoxia as indicated by EF5 staining in the patient-derived xenograft models. **C.** EF5 staining *vs*. tumor growth shows that hypoxia mostly correlates with rapid growth pattern. Tumor growth is defined by the time elapsed between two passages. **D.** Correlation between Ki67 expression and growth rate in xenograft tumors. **E.** Representative sections of double immunofluorescent staining for Ki67 (green) and EF5 (red) of OCIP19, OCIP23 and OCIP51 tumors (DAPI: blue).

The morphological features of the PDX models closely resembled those of the patient donor (Figure 1A). PDX growth pattern, and the levels of hypoxia remained stable over several passages at both the orthotopic and subcutaneous sites (Intraclass Correlation Coefficient = 0.78, Supplementary Figure 2A, 2B). Rather than being correlated with tumor size, the magnitude of hypoxia appeared to be an innate characteristic of individual PDX models that remained stable in the growing tumor (Supplementary Figure 2C). Individual models showed a wide range of hypoxia (4-39%), although the levels for tumors derived from the same patient were relatively consistent independent of the implantation site (Figure 1B). Similarly, these models displayed a range of growth pattern as defined by the time elapsed between two passages (Figure 1C). Ki-67 staining was correlated with tumor growth as expected (*R* = 0.93) (Figure 1D), although a slow growing mucinous tumor OCIP130 (Supplementary Figure 2D, 2E) showed anomalously high levels of Ki-67 staining in cells surrounding the mucin filled ducts (Supplementary Figure 2F). Co-staining of EF5 and Ki-67 showed that the majority of Ki-67 staining was in EF5 negative regions (Figure 1E).

The labeling of hypoxic tissue using 2-nitroimidazole probes like EF5 is dependent on the activity of single-electron reductases, and anomalously low results might occur in tissues deficient in these enzymes. To address this, fresh tissue fragments approximately 1mm^3^ in size were obtained from primary xenografts showing a range of hypoxia (OCIP19, 23, and 51), and incubated in 0.2% oxygen in a hypoxic chamber for 4h in the presence of EF5 as previously described by Koch [28-30]. They were then fixed and processed for immunohistochemistry. As shown in Supplementary Figure 3A, all tumor pieces showed an equal capacity to metabolize EF5 when exposed to low oxygen conditions *ex vivo*, supporting the validity of this technique to measure hypoxia.

### Effects of TH-302 and IR treatment on tumor growth

**Figure 2:**
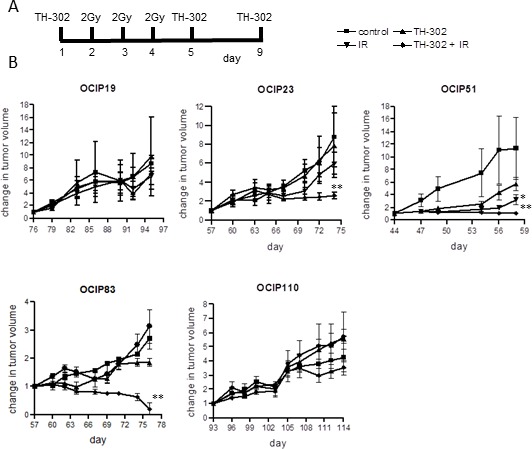
TH-302 treatment reduces tumor weight and growth rate in fast-growing hypoxic xenografts **A.** Treatment schedule for chronic TH-302 treatment of subcutaneous tumors. Animals were treated with 50mg/kg TH-302 on days 1, 5 and 9 and 2Gy of irradiation on days 2, 3 and 4. **B.** Tumor growth of OCIP19 (*n* = 6 per group), OCIP23 (*n* = 6 per group), OCIP51 (*n* = 6 per group), OCIP83 (*n* = 4 per group) and OCIP110 (*n* = 4 per group) in response to treatment with TH-302 or IR alone or with the combination of both according to the treatment schedule. Tumor volume was measured using calipers and referenced back to the starting volume to calculate the change in tumor volume over the course of the treatment. Error bars represent SEM. * p≤0.05; ** p≤ 0.01

Next we examined the effects of treatment with TH-302, which is selectively toxic towards hypoxic cells, and ionizing radiation, which is relatively ineffective under hypoxia, and asked if combined treatment was more effective than the single agents. A protocol was developed combining fractionated radiation with intermittent TH-302 to simulate a clinical treatment regimen, where patients generally receive 2-3Gy per dose (Figure 2A). This treatment, along with appropriate controls, was applied to groups of animals bearing tumors subcutaneously in the hind leg.

Similar to previous observations [25], ionizing radiation (IR) induced DNA damage mainly in EF5-negative cells, assessed by γH2AX staining, whereas damage occurred predominantly in EF5-positive cells following TH-302 treatment (Supplementary Figure 3D). Increased γH2AX labeling was also observed in surrounding oxygenated tissue following treatment with TH-302. This has been previously reported, and attributed to a “bystander effect” [26].

There was considerable variation in the response to treatment. Treatment with either TH-302 alone or in combination with IR had no significant effect on the slow growing, low-hypoxia models OCIP 19 (Figure 2B), OCIP100 and OCIP130 (Supplementary Figure 4A). Treatment of the medium-hypoxia model OCIP23 with TH-302 as a single agent also showed no significant effect, but in combination with IR it delayed tumor growth and significantly reduced the tumor volume (*p* = 0.01) compared to IR alone (Figure 2B). The slow growing hypoxic model OCIP110 showed no response to treatment with TH-302 or IR alone or with the combination, whereas the rapidly-growing hypoxic model OCIP51 was the most sensitive. Treatment with TH-302 or IR alone, lead to a transient delay in the growth of OCIP51 tumors, whereas the combination of TH-302 and IR produced sustained inhibition of tumor growth when compared to IR alone (*p* = 0.034; Figure 2B). At the endpoint there was a significant reduction in tumor size in animals treated with the combination of TH-302 and IR if compared to IR alone (*p* = 0.007), and tumor growth in this treatment group was not re-established at any time during the experiment. Similar results were observed in the low-hypoxic but faster growing model OCIP83. While IR or TH-302 as a single treatment had very little impact on tumor growth (control *vs* IR *p* = 0.81, control *vs* TH-302 *p* = 0.028), the combination of TH-302 and IR resulted in a significant reduction of tumor growth (*p* < 0.0001) (Figure 2B). Indeed, no palpable tumors were observed in part of the animals in this group. No changes in body weight were observed in response to either treatment (Supplementary Figure 4B). Both OCIP23 and OCIP51 tumors treated with either TH-302 or IR showed a highly significant increase in tumor necrosis when compared to the control, that was not observed in OCIP19 and OCIP110 (Supplementary Figure 4C).

### High TIC frequencies correlate with rapid tumor growth

**Figure 3:**
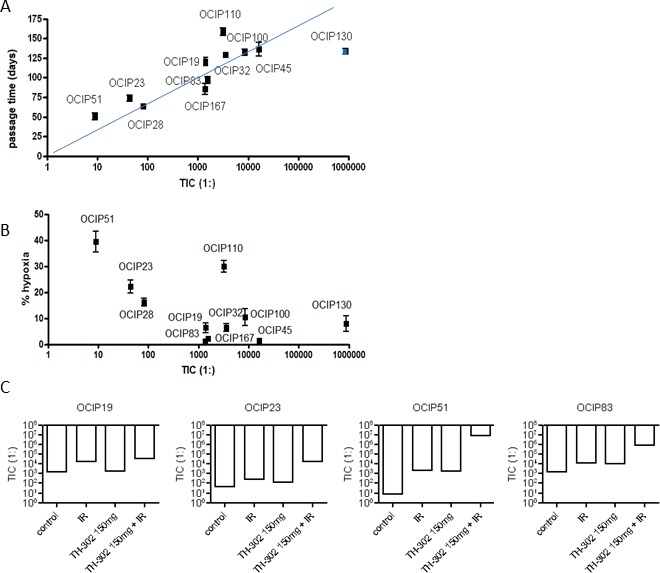
TIC frequency strongly correlates with tumor growth rate but not hypoxia **A.** Statistical analysis shows that TIC frequency strongly correlates with tumor growth (*R* = 0.88), **B.** but not with the magnitude of tumor hypoxia (*R* = 0.4). The apparent rapid growth seen in OCIP130, which had the lowest TIC frequency, is explained by the accumulation of mucin-filled ducts resulting in a tumor mass disproportionate to the cellular content (Supplementary Figure 2E). **C.** Treatment with 10Gy of IR reduced the TIC frequency in most of the tested models. TH-302 as a single agent reduced TIC in a dose dependent manner in most of the tested models with the exception of OCIP23. A similar dose-dependency is observed in the TH-302 + IR group. With the exception of OCIP19, the combination of TH-302 and IR was beneficial compared to IR or TH-302 alone in all tested models.

Tumor-initiating cell (TIC) frequency was measured by limiting dilution assay (LDA) in a total of eleven different models, representing a range of growth rate and hypoxia. We observed a five decade range in TIC frequency between models (Table 1), although the majority had a TIC frequency of 1:1,000 to 1:16,000 which is similar to previous reports for pancreatic cancer stem cells [14]. Tumor morphology as examined by H&E staining remained stable in tumors derived from LDAs when compared to the donor tumor (Supplementary Figure 5). Next we examined the relationships between TIC frequency, hypoxia, and growth rate. The frequency of TIC was strongly correlated with growth rate of the tumors (*R* = 0.89; Figure 3A), but not with their level of hypoxia (*R* = 0.37; Figure 3B).

**Table 1:** Tumor-initiating cell frequency of PDX models

Model	Frequency	lower Frequency	upper Frequency
**OCIP51**	1:8	1:11	1:7
**OCIP23**	1:46	1:67	1:31
**OCIP28**	1:83	1:159	1:43
**OCIP167**	1:1,380	1:2,856	1:666
**OCIP19**	1:1,386	1:2,263	1:849
**OCIP83**	1:1,579	1:3,299	1:756
**OCIP110**	1:3,199	1:6,160	1:1,661
**OCIP32**	1:3,587	1:6,539	1:1,967
**OCIP100**	1:8,447	1:21,391	1:3,335
**OCIP45**	1:16,371	1:35,182	1:7,618
**OCIP130**	1:876,111	1:1,908,670	1:402,149

### Effects of ionizing radiation on tumor-initiating cells

Five models (OCIP19, 23, 51, 83, and 130), representing the ranges of hypoxia, tumor growth, and TIC frequency, were selected to test the effects of TH-302 and IR. Treatment with a single dose of 10Gy significantly reduced TIC frequency in all of the tested models (Table 2, Supplementary Table 1, Figure 3C). TIC frequency was reduced by one order of magnitude in OCIP19, 23 and 83. Similar to the results obtained in the tumor growth experiments (Figure 2), OCIP51 showed the highest sensitivity towards treatment with IR. Although OCIP51 displays the highest magnitude of hypoxia of all models tested (Figure 1C), treatment with IR reduced TIC frequency by three orders of magnitude from 1:8 to 1: 2,014.

**Table 2:** Effects of TH-302 and ionizing radiation on TIC frequency

		Frequency	lower Frequency	upper Frequency
**OCIP19**				
	control	1:1,386	1:2,263	1:849
	IR	1:18,932	1:35,427	1:10,117
	TH-302 50mg	1:162	1:473	1:56
	TH-302 150mg	1:1,639	1:3,600	1:746
	TH-302+IR 50mg	1:4,463	1:9,860	1:2,020
	TH-302+IR 150mg	1:32,225	1:57,178	1:18,162
**OCIP23**				
	control	1:46	1:67	1:31
	IR	1:266	1:431	1:164
	TH-302 50mg	1:107	1:287	1:40
	TH-302 150mg	1:121	1:311	1:44
	TH-302+IR 50mg	1:2,211	1:4,520	1:1,082
	TH-302+IR 150mg	1:18,324	1:38,188	1:8,793
**OCIP51**				
	control	1:8	1:11	1:7
	IR	1:2,014	1:3,368	1:1,204
	TH-302 50mg	1:80	1:131	1:49
	TH-302 150mg	1:1,743	1:3,160	1:962
	TH-302+IR 50mg	1:9,873	1:17,236	1:5,655
	TH-302+IR 150mg	1:7,581,431	1:17,535,190	1:3,277,872
**OCIP83**				
	control	1:1,579	1:3,299	1:756
	IR	1:12,748	1:26,702	1:6,086
	TH-302 50mg	1:810	1:1,589	1:413
	TH-302 150mg	1:9,488	1:24,719	1:3,642
	TH-302+IR 50mg	1:45,903	1:85,665	1:24,597
	TH-302+IR 150mg	1:845,975	1:526,700	1:2,141,477

### Effects of the hypoxia-activated prodrug TH-302 on tumor-initiating cells

Treatment with a single dose of TH-302 reduced TIC frequency in a dose-dependent manner, and this effect was more pronounced in the hypoxic models OCIP23 and OCIP51, compared to OCIP19 and OCIP83 which showed low levels of hypoxia. The combination of either 50mg/kg or 150mg/kg TH-302 and IR was highly effective in both OCIP23 and 51 when compared to the single agents. This effect was particularly striking in OCIP51, where combined treatment with 150mg/kg TH-302 and 10Gy depleted TIC by five orders of magnitude (Table 2, Supplementary Table 1, Figure 3C). Similar to the hypoxic models, addition of TH-302 to IR reduced the TIC frequency of OCIP19 and 83 in a dose-dependent manner, and was considerably more effective compared to TH-302 or IR alone. Comparison of OCIP19 and OCIP83, which differ significantly in growth rate but only marginally in the magnitude of hypoxia (Figure 1), shows higher treatment efficiency in the faster growing model OCIP83. The combination of both 50mg/kg and 150mg/kg of TH-302 with 10Gy of IR lead to a significant reduction of TIC frequencies in OCIP83 when compared to IR alone, while only the higher dose of TH-302 (150mg/kg) led to a significant reduction of TIC frequencies in OCIP83 when compared to IR alone (Table 2). The fifth model tested in these experiments, OCIP130, showed a very low TIC frequency, and as a consequence we were unable to obtain meaningful data testing for TIC depletion with TH-302 or IR.

## DISCUSSION

The PDX models used in this study represent a range of growth characteristics, and show levels of hypoxia similar to those that we observed in pancreatectomy specimens from patients given the hypoxia tracer pimonidazole pre-operatively [31]. The magnitude of tumor hypoxia varied widely between models, and remained similar during tumor growth from first detection at around 0.4 cm in diameter to endpoint of around 1.5cm in diameter. The frequency of TIC spanned five decades, although the majority were in the range 10^3^-10^4^, which is similar to previous reports. There was a strong correlation between TIC frequency and tumor growth, but contrary to expectation we did not observe higher TIC frequencies in the more hypoxic models.

The concept that the hypoxic microenvironment provides a niche supporting TIC survival [7, 15, 19, 20] is of considerable clinical importance, because cells adapted to survive under the adverse conditions associated with hypoxia are predicted to show resistance to radiation treatment and chemotherapy [1-4, 14, 18, 32-35]. Thus they have the potential to repopulate a tumor following initially successful treatment.

We reasoned that if pancreatic cancer TIC reside in a hypoxic niche, then they would be vulnerable to killing by hypoxia-activated prodrugs like TH-302 but relatively insensitive to a single 10Gy treatment with ionizing radiation, whereas the reverse would be true for TIC residing in better oxygenated regions. As summarized in Figure 3, in a subset of four models where this was tested, we found that TH-302 had the greatest effect in the highly hypoxic model OCIP51, and least in OCIP19 which has a low level of hypoxia. In all four models, the combination of 150mg/kg TH-302 + IR gave greater TIC depletion than the single agents. There was an apparent increase in TIC frequency of the low hypoxia model OCIP19 treated with 50mg/kg TH-302, and it remains unclear if this due to an uncharacterized effect of the drug on the TIC assay, or experimental noise. Surprisingly, a single treatment with IR significantly reduced the number of TIC in all tested models, suggesting that these cells exist in well oxygenated as well as hypoxic microenvironments.

However, it should be noted although tumors established at the limiting dilution, which are assumed to be derived from a single cell (or very low number of cells), appear to recapitulate the phenotypic features of the parent tumor, this does not prove that they are true stem cells. Therefore these experiments do not exclude the possibility of a hypoxic stem cell niche in pancreatic cancers.

The magnitude of tumor hypoxia was highly consistent between replicate tumors from the same patient, similar to our earlier finding [11] suggesting an underlying genetic (or epigenetic) basis to explain the large variation in the levels of hypoxia between the individual PDX. Nevertheless, at the present time it is not clear if the development of hypoxia is simply the consequence of rapid proliferation pushing cells away from well vascularized regions, or if hypoxia is playing a more active role in biological aggression, as suggested by several recent studies including those from our own group [5-13, 36]. This is being addressed in our ongoing pimonidazole study (NCT01248637) that will seek for candidate genomic signatures in a total of 100 pancreatectomy specimens. Finally, we note that the combination of IR and 150mg/kg TH-302 was strikingly effective controlling the growth of some PDX. Local disease control remains a major problem in the clinic, and radiation therapy has a role both in the pre-operative neoadjuvant setting, and as palliation for unresectable disease. However, even with current precision-guided protocols, the benefit is limited by the insensitivity of the tumor relative to adjacent small bowel. Hypoxia has long been recognized as a major cause of radiation resistance, and in theory could become more important with the introduction of stereotactic body radiotherapy (SBRT) protocols that deliver treatment in small numbers of high dose fractions that provide less time for tumor reoxygenation to occur. Using positron-emission tomography with the 2-nitroimidazole tracer ^18^F-fluoroazomyin arabinoside (^18^F-FAZA), we recently showed that pancreatic cancers have a wide range in the levels of hypoxia [37]. Our current results suggest that there might be considerable benefit incorporating hypoxia-targeted agents into clinical radiation therapy protocols, and additional benefit using non-invasive techniques to stratify patients on the basis of tumor hypoxia.

## MATERIALS AND METHODS

### Primary patient-derived xenografts

Patient-derived xenografts (PDX) were established from pancreatectomy samples as previously described [11, 12], according to institutional guidelines for human and animal research. For TIC experiments, subcutaneous tumors were established by preparation of single-cell suspensions as previously described [11], and injected into the flanks of recipient mice. To measure tumor growth inhibition, xenografts were established subcutaneously [11]. Subcutaneous tumors were measured using callipers, and volume calculated according to the formula width^2^×length×0.5.

### Limiting dilution assay

This was done using tumors grown to a diameter of approximately 1cm. Single-cell suspensions were prepared, and a sample was stained using anti-mouse CD31-FITC and H2K-FITC antibodies to establish the mouse cell content. Pr*opidium iodide* was added to label dead cells, and the percentage of viable cancer cells counted using a hemocytometer. As illustrated in Supplementary Figure 1, 3-6 dilutions of human cells per sample were injected into the flanks of three pre-irradiated (2Gy) NOD/SCID mice per dilution. Tumor take rate was observed over the course of 6 months. TIC frequency was calculated using the L-Calc software (Stem Cell Technologies, Vancouver, BC) using a minimum of two replicate experiments.

### Histological analysis

To determine the level of tumor hypoxia, mice were injected intraperitoneally (ip) with the 2-nitroimidazole EF5, 30mg/kg, 3h prior to sacrifice [28-30]. Tumors were excised, fixed and paraffin-embedded, and tissue sections stained using an anti-EF5 antibody provided by Dr. Koch, as previously described [28]. Slides were scanned at 20x resolution using an Aperio Scanscope XT scanner, and the percentage of pixels stained for EF5 determined using the Aperio Imagescope software (Vs.11.1.2.752, Aperio Technologies, Vista, CA).

Dual immunofluorescence staining was used to measure γH2AX and Ki-67, in relation to hypoxia. For combined γH2AX plus EF5 labelling, sections were incubated with γH2AX antibody (1:1000; Bethyl, Montgomery, TX) and EF5-CY5 at room temperature overnight. For dual Ki67 plus EF5 staining, sections were incubated sequentially at room temperature with a rabbit anti-Ki67 antibody (1:1000; Novus-Biologicals, Littleton, CO) for 2h and EF5-CY5 overnight, then with secondary anti-rabbit antibody for 1h. Sections were counterstained with DAPI, and air dried. Image acquisition was done using a TissueScope-4000 laser scanning system (Huron Technologies, Waterloo, ON) at 1μm/pixel using violet, blue and red laser excitation. Then the slides were re-stained for H&E, and imaged using the instrument's transmitted light mode to capture a brightfield image that was aligned with the corresponding fluorescence image using Adobe Photoshop, and image analysis done using Definiens TissueStudio (Munich, Germany). Hypoxia, proliferation and DNA damage were evaluated in the viable tissue by first developing a training set with the H&E images to identify and segment regions of interest, including tumor, necrosis, and stroma, and to exclude artefacts. Automated analysis was then performed within the tumor region, with individual nuclei segmented using the DAPI image.

### Treatments with IR and hypoxia-activated prodrug TH-302

TH-302 was provided by Threshold Pharmaceuticals (South San Francisco, CA), and formulated in saline at 5mg/ml. Irradiation treatment was performed using a Cs^137^ irradiator (Gammacell-40, Best Therapeutics, Chalk River, Canada) at a dose rate of approximately 0.8Gy/min.

To test effects on tumor growth, animals were treated when the volume reached approximately 100mm^3^. Preliminary experiments were done testing the effects of IR and TH-302 in the OCIP51 model. Based on these, the combination treatment protocol was developed. Animals were treated with 50mg/kg TH-302 injected ip on days 1, 5 and 9 and/or 2Gy irradiation on days 2, 3 and 4. Animals were immobilized for the irradiation in a Plexiglas container and shielded with lead so that irradiation was only delivered to the lower half of the animals. Tumor volumes was monitored three times a week and animals were euthanized when tumors reached the humane endpoint.

For LDA animals were treated with a single dose of either 50mg/kg or 150mg/kg TH-302 injected ip or saline, or with 10Gy. For the combination treatments, animals received TH-302 10min prior to 10Gy. Tumors were excised 12h after treatment and processed for LDA. To examine the acute effect of TH-302 treatment, animals were treated with one dose of 50mg TH-302 or 10Gy, the combination of TH-302 and IR or saline. Tumors were excised 12h or 24h after treatment and processed for image analysis.

### Statistical analysis

EF5 staining was analysed using non-parametric statistics. The overall differences between the four treatment groups were assessed using the Kruskal-Wallis test, and for the differences between two treatments the Mann-Whitney test was utilized. To assess the associations between TIC, growth and hypoxia, Spearman correlation coefficients were calculated.

## SUPPLEMENTARY MATERIAL TABLE AND FIGURES


