# Supplementary material for: Targeting hypoxic microenvironment of pancreatic xenografts with the hypoxia-activated prodrug TH-302
Source: Oncotarget. 2016 May 29;7(23):33571–80. doi: 10.18632/oncotarget.9654 (PMC5085103; doi:10.18632/oncotarget.9654)
Supplement: Supplementary file 1 [file oncotarget-07-33571-s001.pdf]

Targeting hypoxic microenvironment of pancreatic xenografts with the hypoxia-activated prodrug th-302

Supplementary Material

Supplementary Table 1: LDA raw data for OCIP51

cell number	10^7*	10^6	10^5	10^4	10^3	10^2	$5x10^1$	10^1	5
**control**						12/12	12/12	12/12	3/10
**IR**				12/12	6/12	1/12			
**TH-302 50mg/kg**				12/12	12/12	11/12	3/6	1/6	
**TH-302 150mg/kg**		6/6	5/6	5/12	3/12	0/12		0/6	0/6
**IR + TH-302 50mg/kg**		8/8	12/12	7/12	2/12	0/6			
**IR + TH-302 150mg/kg**	3/6	4/12	0/6	0/6					

* take rate / total injected

A

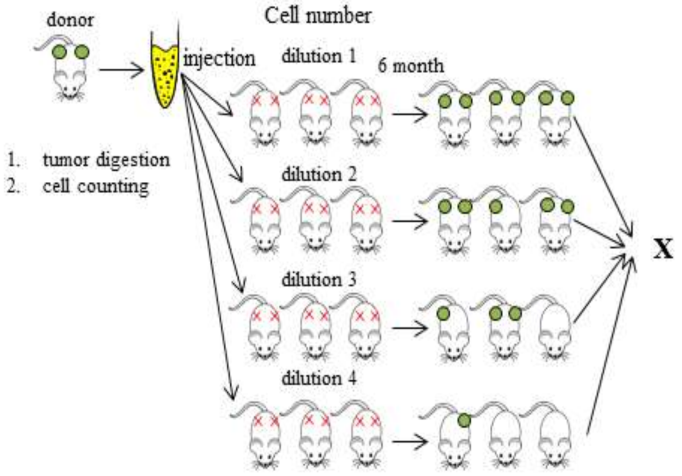

B

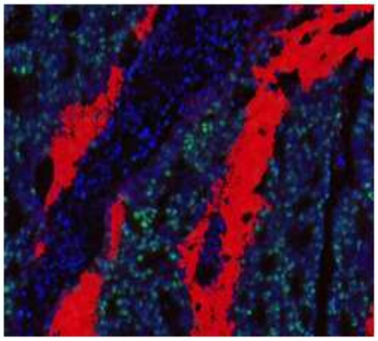

C

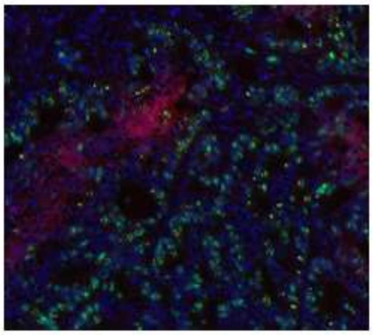

Supplementary Figure 1: Schematic overview of the limiting dilution assay.

(A)Xenograft tumors were mechanically minced and enzymatically digested to achieve a single cell suspension. The number of tumor cells was established and different dilutions of tumor cells were re-injected into NOD/SCID mice. The formation of tumors was monitored over the course of 6 months. TIC frequency was calculated using the L-Calc software. (B) Hypoxia and (C) proliferation were evaluated in the viable tissue by first developing a training set with the H&E images to identify and segment regions of interest, including tumor, necrosis, and stroma, and to exclude artefacts.

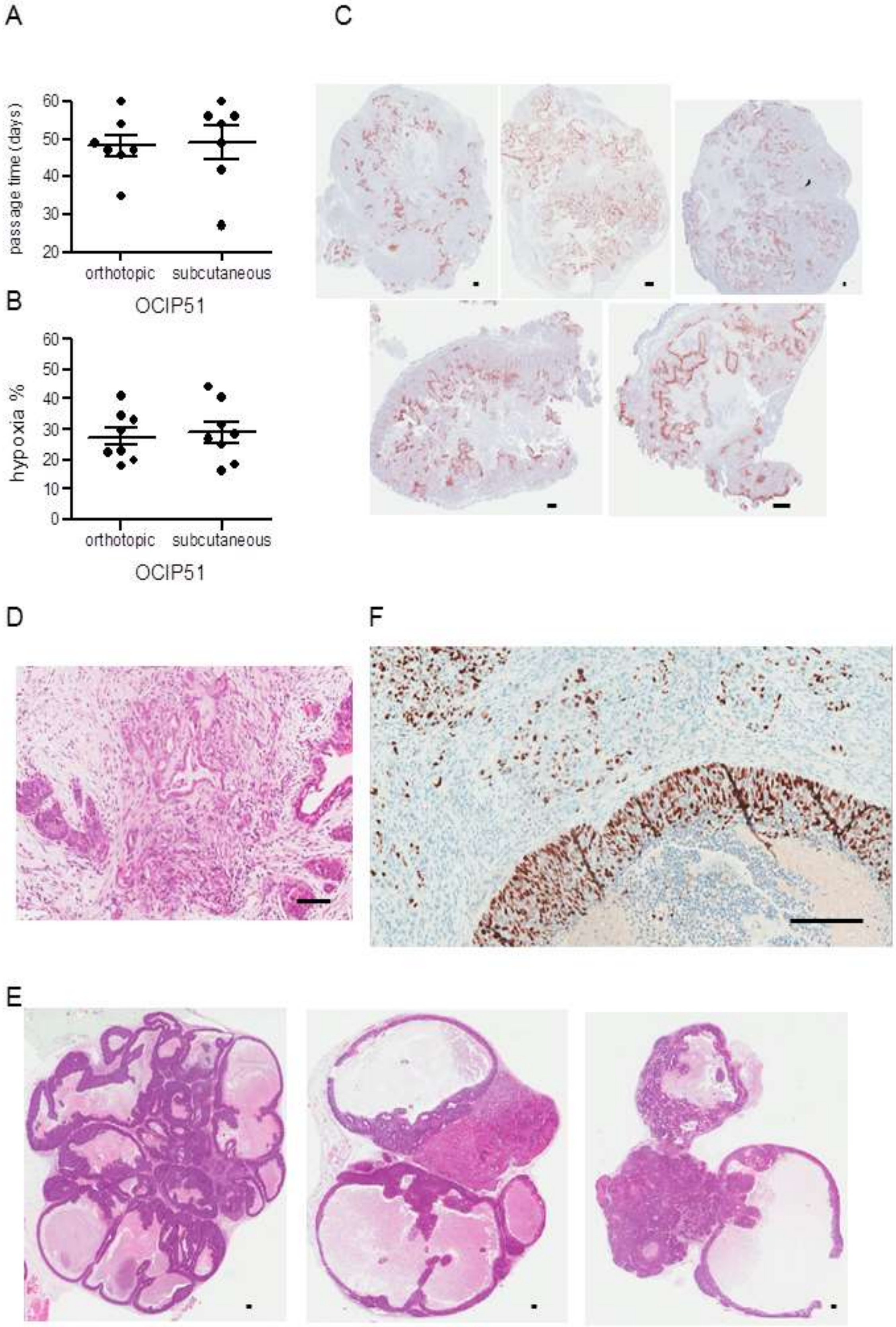
A
passage time (days)
60
50
40
30
20
orthotopic
subcutaneous
OCIP51
B
hypoxia %
60
50
40
30
20
10
0
orthotopic
subcutaneous
OCIP51
C
D
F
E

Supplementary Figure 2: Characterization of the patient-derived xenograft models.

As shown on the example of OCIP51, the patient-derived xenograft models showed stable (A) growth pattern and (B) hypoxia levels over several passages in NOD/SCID mice at both the orthotopic and subcutaneous sites. (C) Representative sections of OCIP51 xenograft tumors stained for EF5. The sections are derived from differently sized tumors. The black bar indicates 100µm. (D) H&E stained sections of the OCIP130 patient specimen. (E) Representative H&E stained sections of the OCIP130 xenograft. Mice bearing OCIP130 had to be sacrificed early due to the formation of large cysts. (F) Representative Ki67 stained sections of the OCIP130 xenograft model.

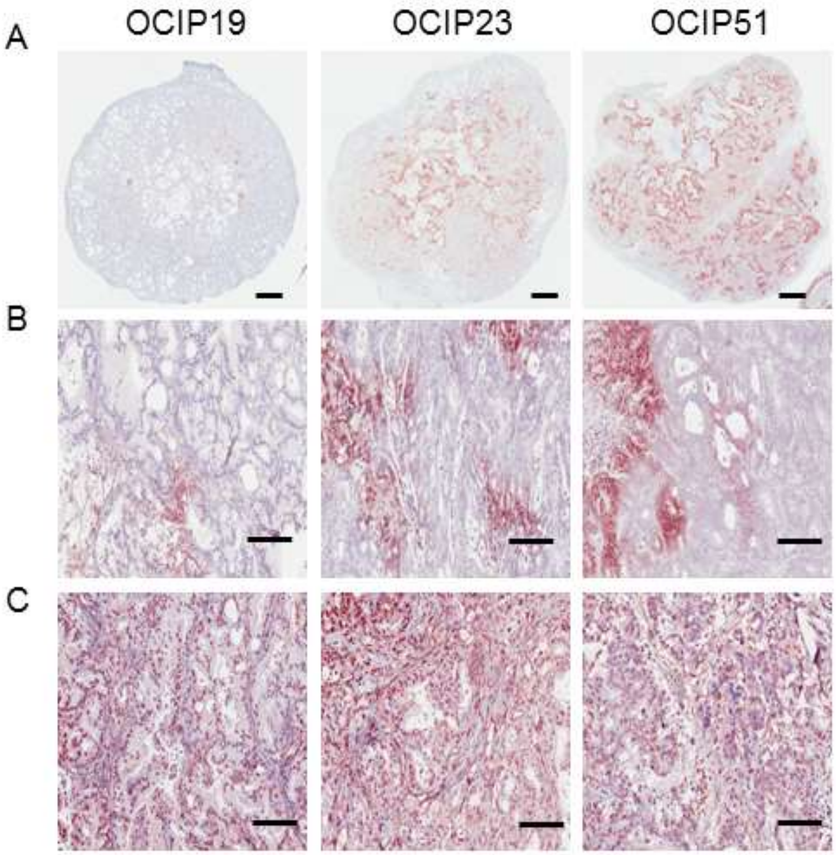
A
OCIP19
OCIP23
OCIP51
B
C

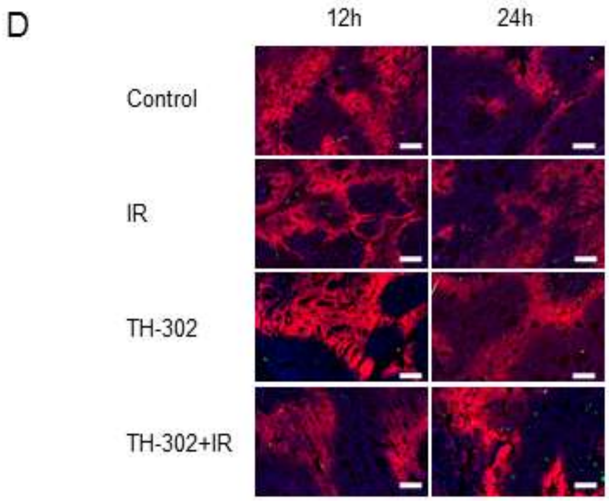
D
12h
24h
Control
IR
TH-302
TH-302+IR

Supplementary Figure 3: EF5 staining is representative of the magnitude of tumor hypoxia. Representative sections of (A) whole OCIP19, OCIP23 and OCIP51 tumors or (B) at 10x magnification. (C) Representative images of tumor pieces of OCIP19, OCIP23 and OCIP51 tumors incubated *in vitro* with EF5 under hypoxic conditions. (D) Representative section of double immunofluorescent staining for γH2AX (green) and EF5 (red) of OCIP51 tumors treated with either TH-302 or IR alone or with the combination of TH-302 + IR. IR induced DNA damage mainly in EF5-negative cells whereas damage occurred predominantly in EF5-positive cells following TH-302 treatment (DAPI: blue). Consistently, DNA damage was present in both the oxic and the hypoxic microenvironment after treatment with the combination of TH-302 and IR.

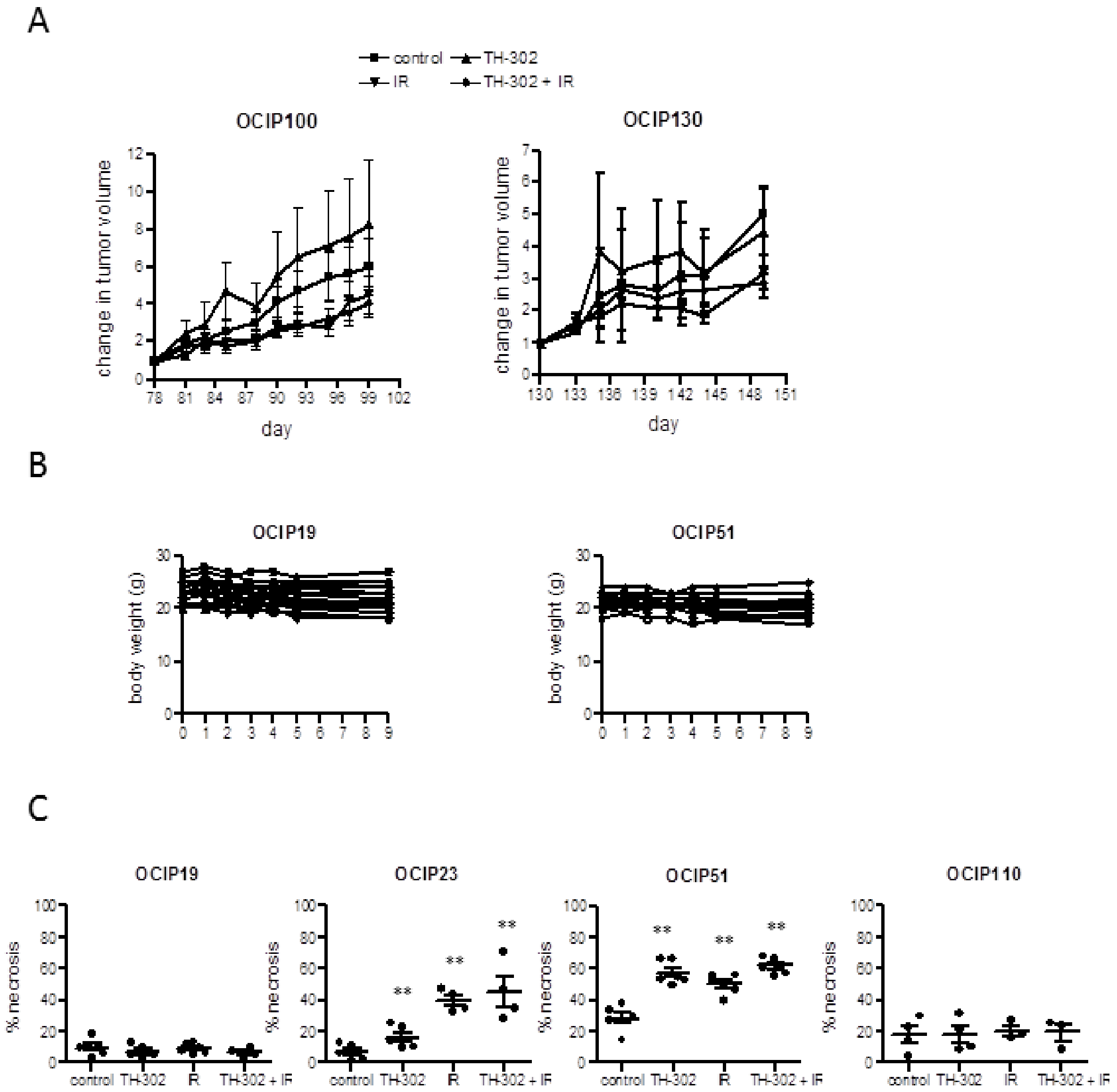

Supplementary Figure 4: Treatment with TH-302+IR reduces tumor growth in fast-growing hypoxic tumors.

(A) Tumor growth of OCIP100 (n=4 per group), and OCIP130 (n=4 per group) in response to treatment with TH-302 or IR alone or with the combination of both according to the treatment schedule shown in Figure 2A. (B) Weight of animals bearing OCIP19 and OCIP51 tumors over the course of the treatment cycle. (C) Quantitative analysis of the necrotic fractions in tumors of

OCIP19, OCIP23, OCIP51 and OCIP110 in H&E stained sections (OCIP23: TH-302 $p=0.002$, IR $p=0.006$, TH-302+IR $p=0.006$; OCIP51: TH-302 $p=0.008$, IR $p=0.004$, TH-302+IR $p=0.002$). Error bars represent SEM. * $p \leq 0.05$; ** $p \leq 0.01$

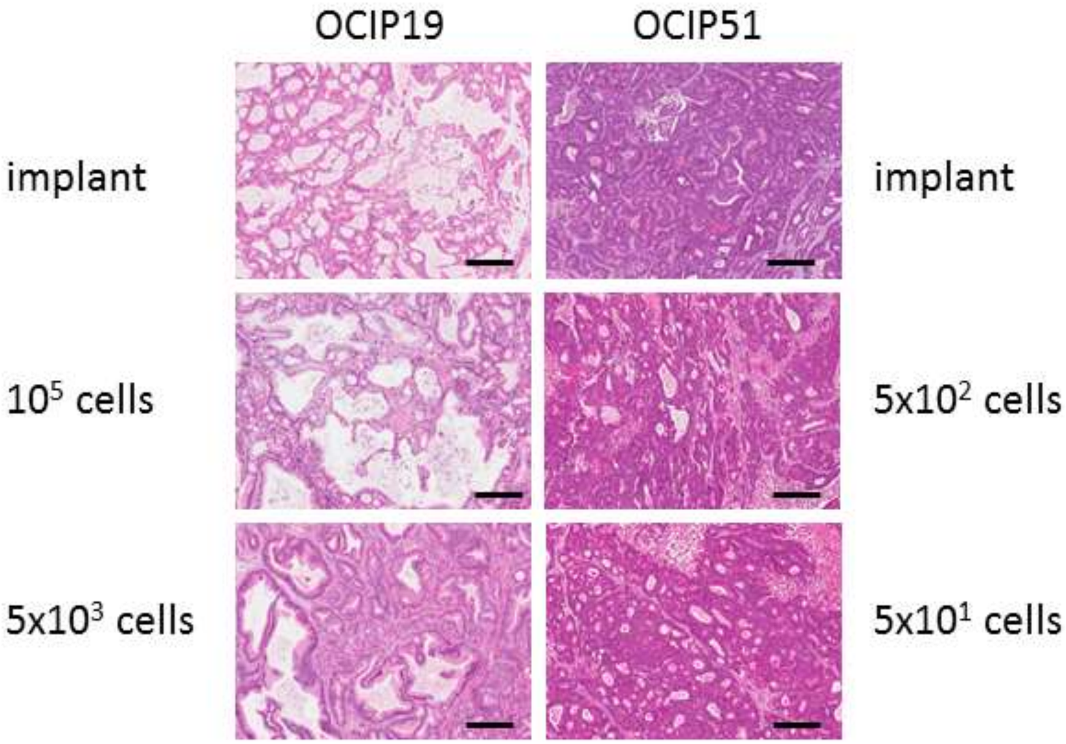

Supplementary Figure 5: Tumors established from limiting dilutions closely resemble the donor tumors.

Representative H&E stained sections of OCIP19 and 51 tumors derived from tumors implanted subcutaneously and limiting dilution.
